# No association between the progesterone receptor gene polymorphism (+331G/a) and the risk of breast cancer: an updated meta-analysis

**DOI:** 10.1186/s12881-017-0487-3

**Published:** 2017-10-30

**Authors:** Xing-ling Qi, Jun Yao, Yong Zhang

**Affiliations:** 1 Cancer Hospital of China Medical University, Liaoning Cancer Hospital & Institute, Shenyang, 110042 People’s Republic of China; 2 School of Forensic Medicine, China Medical University, Shenyang, 110122 People’s Republic of China; 3 No. 44 Xiaoheyan Road, Dadong District, Shenyang, 110042 People’s Republic of China

**Keywords:** Progesterone receptor, + 331G/a, Breast cancer, Meta-analysis

## Abstract

**Background:**

Many published studies have estimated the association between the +331G/A (rs10895068) polymorphism in the progesterone receptor (PgR) gene and breast cancer risk. However, the results remain inconsistent and controversial. To address this inconsistency, we systematically interrogated the aforementioned association via a meta-analysis.

**Methods:**

Through a literature search, we identified 13 case-control studies, including 12,453 cases and 14,056 case-free controls. The strengths of reported associations were evaluated using odds ratios (ORs) with 95% confidence intervals (95%CIs).

**Results:**

An association was found between +331G/A polymorphism and +331G/A risk in the dominant model (*p* = 0.027). Via subgroup analysis, we found no association between +331G/A and breast cancer risk in Caucasians, Asians or mixed racial groups.

**Conclusions:**

Through meta-analysis, we were able to gain insight into previously reported associations between +331G/A polymorphism and breast cancer risk. However, further studies are still needed to provide more evidence.

## Background

The most common malignant neoplasm in women, breast cancer has a higher developed versus developing countries. It is a complex and multi-factorial disease caused by a combination of genetic and environmental factors. Although the exact mechanism of breast cancer carcinogenesis is still not completely elucidated, many factors are known to influence its development including age, nulliparity, early menarche, late menopause, and family history [1]. In addition, inherited susceptibility accounts for approximately 27% of breast cancer risk, demonstrating that genetic factors contribute to risk of developing breast cancer [2].

Progesterone (PR) is known to regulate cell proliferation and differentiation in the female reproduction system [3]. Dysregulated oestrogen and progesterone signaling results in disorders such as breast cancer, subfertility, endometriosis, and endometrial cancer that depend on steroid hormones [4]. Negative associations between PR protein levels and pathological grade, tumor size, and axillary lymph node involvement are frequently reported [5–8]. Additionally, PR positive tumors are believed to confer a more favorable prognosis. Moreover, primary breast tumors which lack PR are more prone to develop secondary sites than tumors which express PR in those postmenopausal patients [9]. This suggests that PR may also limit breast cancer progression.

The progesterone receptor (PgR) is essential for mediating the effects of progesterone, which is necessary to establish and maintain pregnancy. The PgR gene encodes two iso-forms, PR-A and PR-B. Breast cancers commonly express a predominance of one PR isoform, and the loss of coordinated expression in the ratio between PR-A/PR-B proteins within a cell is likely to result in an aberrant hormonal response [10]. The PgR gene contains eight exons and seven introns (A-G), and is located on chromosome 11q22–23 [11]. While associations between PgR gene polymorphisms and breast cancer have been well-studied, results remain inconsistent [12–15].

Among the variations of PgR gene, the +331G/A variant (rs10895068), locating in the promoter region, has been wildly studied. One case–control study including 990 cases and 1364 controls showed that the +331G > A polymorphism increases PR-B isoform expression, which is reported to increase PR-B-dependent mammary cell proliferation, thereby promoting breast cancer [16]. However, no association was found between +331G > A and breast cancer risk in a recent study of postmenopausal women [14]. Although a biological mechanism is plausible, the role of the +331G > A polymorphism in breast cancer remains ambiguous. We hypothesize that conflicting results are due to the limited sample sizes as well as differing genetic backgrounds. Meta-analysis can be used as a statistical method to reconcile studies with inconsistent results [17]. Therefore, we employed this method to investigate the relationship between the PrG +331G/A polymorphism and breast cancer risk.

## Methods

### Selection of eligible studies

We used four online electronic databases to select studies to include in this meta-analysis (PubMed, Web of Science, and Embase in English and China National Knowledge Infrastructure Database in Chinese; most recent search update, February 2017). Search terms included “breast cancer” or “breast neoplasm” or “mammary” combined with “progesterone receptor gene” or “PgR” or “+331G/A” or “+331G > A” or “rs10895068” and with “polymorphism” or “variant” or “genotype” or “allele”, without any limitation applied. Referenced lists of all included studies were then manually searched to identify any additional eligible studies. Only the study with the most recent, complete data was included when multiple studies included the same set of subjects.

### Inclusion and exclusion criteria

Included studies met the following criteria: (1) case-control design; (2) clinical trial evaluating associations between +331G/A gene polymorphisms and breast cancer susceptibility; (3) pathological confirmation of breast cancer diagnosis was reported for all patients; (4) data regarding sample size and individual genotype frequencies were available for all cases and controls; and (5) at least two comparison groups (cancer group and control group) were included. Exclusion criteria: (1) duplication of prior studies and (2) meta-analysis, letters, reviews, or editorial articles.

### Data extraction

Two investigators (Xing-ling Qi and Jun Yao) independently extracted data from eligible studies. Inconsistencies were resolved via discussion between the investigators. We recorded the first author’s name, publication year, country of origin, ethnicity studied, sample size, genotypes and allele frequencies for patients with the PgR +331G/A polymorphism, and Hardy-Weinberg equilibrium (HWE) results for controls groups. We recorded studies including more than one ethnicity as mixed ethnicity.

### Statistical analysis

We used STATA 12.0 software (Stata Statistical software, College Station, TX, USA) to perform all statistical analysis. PRISMA checklists and guidelines were adhered to when performing the meta-analysis [18]. For control groups, we used Chi-square tests to analyze the Hardy-Weinberg equilibrium (HWE), with *p* < 0.05 indicating a significant deviation. Pooled frequency analyses were performed using Thakkinstian’s method [18, 19]. The strength of associations between the +331G/A polymorphism and breast cancer risk were evaluated using odds ratios (ORs) and 95% confidence intervals (CIs). Two-tailed tests were used to generate all *p* values.

We used five models to evaluate associations the +331G/A and breast cancer risk: allele model, dominant model, recessive model, homozygote comparison model, and heterozygote model. A random effects model was used to pool effect sizes of all included studies for a possible effect size across populations with different genetic backgrounds after considering the heterogeneity among the included studies [20]. We also used A as the risk allele to compare OR1 (AA vs. aa), OR2 (Aa vs. aa), and OR3 (AA vs. Aa) and further determined the genetic model that was the most appropriate under the instruction, as previously described [21, 22]. Heterogeneities among studies were estimated using an I^2^ test, and describe I^2^ values as low (25%), moderate (50%), or high (75%) estimates [22]. A Z-test resulting in a *p* value less than 0.05 determined statistical significance. We also explored the effect of included studies on combined ORs via sensitivity analysis employing sequential omission of each study. In addition, we conducted subgroup analyses by ethnicity (i.e. Caucasian, Asian, and mixed races) as well as by source of control subjects (i.e. hospital-based vs. population-based). We generated a funnel plot to reflect any possible publication bias [23, 24], with an Egger’s test resulting in a*p* < 0.05 indicating significant publication bias.

## Results

We performed the online search of multiple databases for available studies reporting associations between PgR +331G/A polymorphisms and breast cancer risk. We included 13 original articles in this meta-analysis after meeting inclusion criteria. As shown in Table 1, the studies eventually involved 12,453 patient and 14,056 control subjects [12, 14, 16, 25–33]. The frequencies of each genotype and allele along with their HWE values were described in Table 2. All studies reported control genotype distributions in accordance with HWE, save for that of Kotsopoulos, et al. (2009) (*p* < 0.0001) [25].

**Table 1:** Baseline characteristics of qualified studies in this meta-analysis

Author	Year	Country	Ethnicity	Controls source	Mean age of control group	Cases, n	Controls, n
De Vivo	2003	America	Caucasian	hospital-based	57.2	990	1364
Diergaarde	2008	America	Caucasian	population-based	–	323	650
Feigelson	2004	America	Caucasian	population-based	62	479	494
Fernandez	2006	Spain	Caucasian	population-based	–	544	553
Huggins	2006	America	Caucasian	hospital-based	–	1298	1728
Jin	2008	China	Han	population-based	48.67	206	214
Johnatty	2008	Australia	Caucasian	population-based	–	1443	530
Kotsopoulos	2009	America	Caucasian	hospital-based	–	1664	2391
Pearce	2005	America	Caucasian	population-based	–	1674	2432
Pooley	2006	Norfolk	Caucasian	population-based	–	2187	2269
Reding	2009	America	mixed race	population-based	–	1264	1021
Romano	2005	Netherlands	Caucasian	population-based	64.8	535	379
Romano	2007	Netherlands	Caucasian	hospital-based	–	169	31

**Table 2:** Distribution of genotype and allele frequencies of the *PGR* + 331G/A variation

	Genotype distribution								Allele frequency				
	Cases, n				Controls, n				Cases, %			Controls, %	
Author	GG	AG	AA		GG	AG	AA	*P*_HWE_	G	A		G	A
De Vivo	864	126*			1218	139*		–	–	–		–	–
Diergaarde	294	29*			580	70*		–	–	–		–	–
Feigelson	425	53	1		445	48	1	0.8039	94.3	5.7		94.9	5.1
Fernandez	508	36	0		509	43	1	0.9266	97.0	3.0		96.0	4.0
Huggins	1134	164*			1560	168*		–	–	–		–	–
Jin	182	24	0		199	15	0	0.5952	94.0	6.0		96.0	4.0
Johnatt	1282	161*			474	56*		–	–	–		–	–
Kotsopoulos	1463	195	6		2174	202	15	<0.0001	94.0	6.0		95.0	5.0
Pearce	1596	76	2		2317	113	2	0.6086	97.6	2.4		97.6	2.4
Pooley	1929	253	5		2002	260	7	0.6379	94.0	6.0		94.0	6.0
Reding	1128	161*			910	111*		–	–	–		–	–
Romano	476	48	11		339	37	3	0.0874	93.0	7.0		94.0	6.0
Romano	153	15	1		25	5	1	0.2781	95.0	5.0		88.7	11.3

*P*_HWE_ the *P* value of Hardy-Weinberg equilibrium test in the genotype distribution of controls, *For these just presenting the genotyping of AG + AA, dominant model is calculated only

### Association between PgR +331G > A and breast cancer

**Fig. 1:**
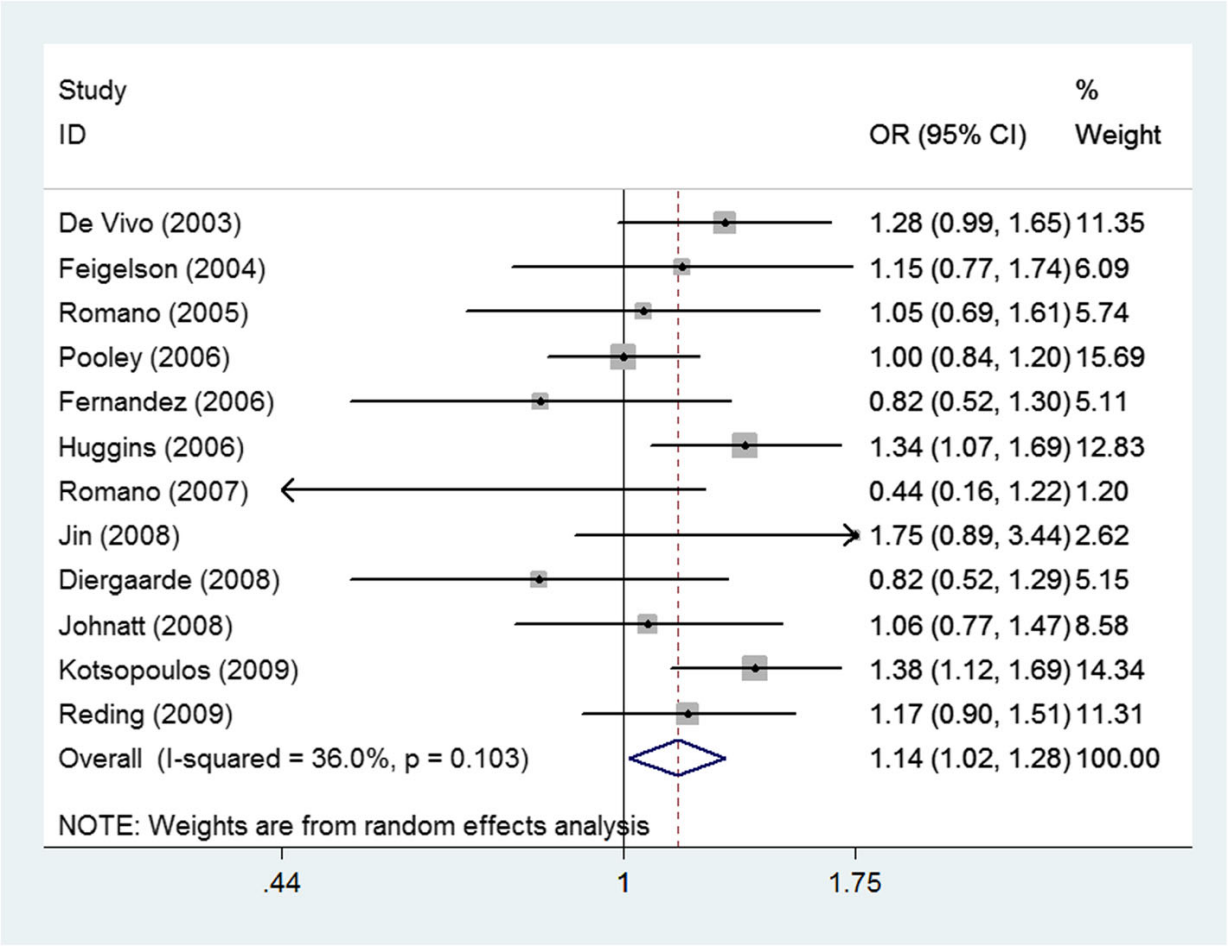
Forest plot of the association between +331G/A in the *PGR* gene and breast cancer in a dominant model (AG + AA vs. GG)

Table 3 shows our results generated using five genetic models to evaluate associations between the +331G > A polymorphism and breast cancer risk. Genetic model selection principles were used to determine the dominant model. Our summary results indicate that an association is indeed present between PgR +331G > A and the risk of breast cancer. Using a random effects model, we calculated a pooled OR of 1.140 (*p* = 0.027, 95% CI = 1.015–1.279) (Fig. 1).

**Table 3:** Summarized ORs with 95% CIs for the association between *PGR* polymorphism and breast cancer

Polymorphism	Genetic model	n	Statistical model	OR	95% CI	p_z_	I^2^ (%)	p_h_	p_e_
+331G/A									
	Allele contrast	8	Random	1.073	0.915–1.257	0.388	43.9	0.086	0.871
	Homozygous codominant	8	Random	0.863	0.488–1.524	0.611	0	0.479	0.937
	Heterozygous codominant	8	Random	1.084	0.908–1.294	0.374	48.4	0.06	0.767
	Dominant	12	Random	1.140	1.015–1.279	0.027	36.0	0.103	0.686
	Recessive	8	Random	1.084	0.658–2.277	0.374	48.5	0.059	0.774

*n* the number of studies, *p*_z_ *P* value for association test, *p*_h_, *p* value for heterogeneity test, *p*_e_ *p* value for publication bias test

### Subgroup analysis

We found no association between +331G/A polymorphism and breast cancer risk in Caucasian (*p* = 0.102, OR = 1.116, 95% CI = 0.978–1.272,), Asian (*p* = 0.105, OR = 1.749, 95% CI = 0.890–3.438) and mixed race (*p* = 0.231, OR = 1.170, 95% CI = 0.905–1.513) populations via subgroup analysis. Furthermore, using subgroup analysis by source of controls, there was an association between +331G/A locus and breast cancer risk in hospital-based (*p* = 0.004, OR = 1.295, 95% CI = 1.087–1.543,), but not in population-based controls (*p* = 0.440, OR = 1.046, 95% CI = 0.934–1.171; Table 4).

**Table 4:** Stratified analysis of the association of *PGR* polymorphism with breast cancer under dominant model

Subgroup analysis	+331G/A					
	n	OR	95% CI	p_z_	I^2^ (%)	p_h_
Overall	12	1.140	1.015–1.279	0.027	36.0	0.103
Ethnicity						
Caucasians	10	1.116	0.978–1.272	0.102	42.6	0.074
Han	1	1.749	0.890–3.438	0.105	–	–
mixed race	1	1.170	0.905–1.513	0.231	–	–
Source of controls						
Population-based	8	1.046	0.934–1.171	0.440	0.0	0.586
Hospital-based	4	1.295	1.087–1.543	0.004	36.2	0.195

*n* the number of studies, *p*_z_ *p* value for association test, *p*_h_ *p* value for heterogeneity test

### Sensitivity analysis

We examined the influence of individual studies the pooled ORs for +331G/A via sensitivity analysis involving omitting each study in each genetic model; the results did not change. This indicates that our results are statistically robust for all five genetic models examining associations between +331G/A and breast cancer susceptibility.

### Publication bias

**Fig. 2:**
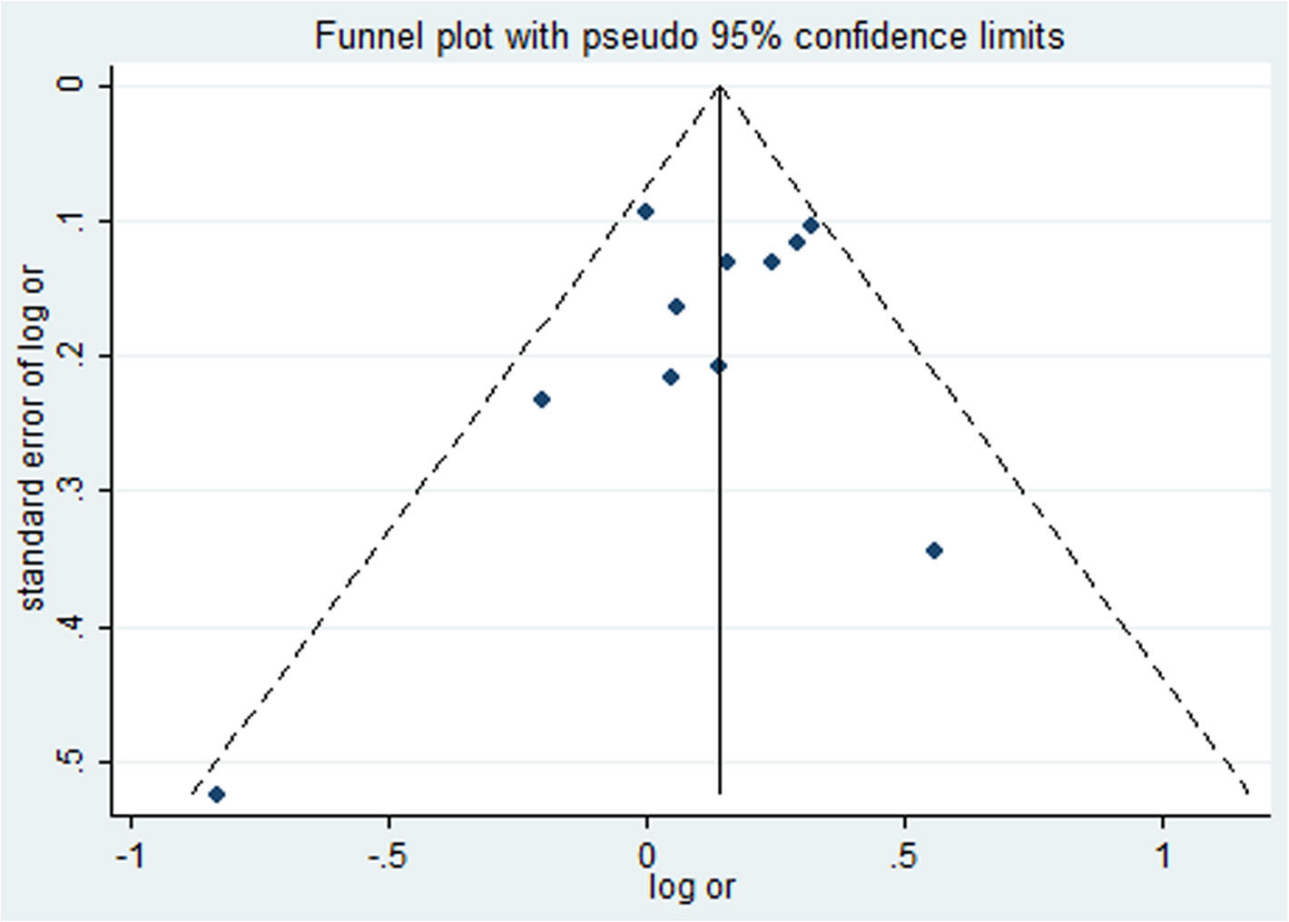
Funnel plot for evaluation of publication bias in breast cancer

We assessed possible publication bias using a Begg’s funnel plot and Egger’s test. As shown in Fig. 2, no obvious asymmetry was observed in the funnel plot all genotypes in the overall population, and Begg’s test results did not reveal any publication bias (*p* > 0.05).

## Discussion

This meta-analysis included 12,453 breast cancer cases and 14,056 controls, and was used to evaluate reported associations between breast cancer risk and the +331G/A (rs10895068) functional polymorphism in the PgR gene promoter. In the dominant model, when all studies meeting eligibility criteria were pooled, we found an association between +331G/A and breast cancer risk. However, after subgroup analysis, this association disappeared in Caucasians, Asian, and mixed race. Therefore, we could cautiously assert that there is no association of the +331G/A PgR gene polymorphism and breast cancer susceptibility in Caucasian and Asian populations.

There have been several prior meta-analysis studies reporting on this particular association, with mixed results. An association between breast cancer risk and PgR +331G/A was reported by Yang, et al. [34]. However, the other two published meta-analyses, which each included more studies than that of Yang, et al., did not confirm this association [35, 36]. The present study, however, has several advantages over these prior studies. First, more recently-published studies were included in the present meta-analysis, which may underscore the reliability of our findings. Second, the present study added additional subgroup analyses by both ethnicity and sources of controls to control for heterogeneity. Third, we also included a Chinese database in our literature search to more comprehensively assess studies in Chinese populations. These advantages allowed us to more precisely assess the + 331G/A *PgR* gene polymorphism and breast cancer risk associations than previous meta-analyses.

There were several limitations to this study that may have affected our results. Firs, only 13 studies were included in our meta-analysis, which limited subsequent analyses because of a shortage of original studies. Second, there was moderate heterogeneity in the overall meta-analysis and in the subgroup analysis that suggested that ethnicity and source of controls, to some extent, contributed heterogeneity between studies. Third, other factors influencing breast cancer, such as genetic background, environment, and lifestyle factors, should also be considered. Finally, there was only ten studies that specified Caucasians and just one study that compared certain populations (Asian and mixed race) in the ethnicity sub-group analyses. Thus, the discrepancy of association among different ethnic sub-groups should be interpreted carefully.

## Conclusion

In conclusion, our meta-analysis suggested that the +331G/A polymorphism may not be associated with susceptibility to breast cancer. However, because of the comparatively insufficient number of published studies included, our conclusions require support from additional studies. More evidence from epidemiologic studies is required to validate our results regarding the role of +331G/A (rs10895068) in the genetic susceptibility to breast cancer.

## Abbreviations

CIs: confidence interval
HWE: Hardy-Weinberg equilibrium
ORs: Odds ratios
PgR: progesterone receptor
PR: Progesterone
